# Understanding the Needs of Young Women Regarding Breast Cancer Risk Assessment and Genetic Testing: Convergence and Divergence among Patient-Counselor Perceptions and the Promise of Peer Support

**DOI:** 10.3390/healthcare4030035

**Published:** 2016-06-28

**Authors:** Chalanda Evans, Rebekah J. Hamilton, Kenneth P. Tercyak, Beth N. Peshkin, Kantoniony Rabemananjara, Claudine Isaacs, Suzanne C. O’Neill

**Affiliations:** 1 Lombardi Comprehensive Cancer Center, Georgetown University, 3300 Whitehaven Street, NW, Suite 4100, Washington, DC 20007, USA; cne8@georgetown.edu (C.E.); tercyakk@georgetown.edu (K.P.T.); peshkinb@georgetown.edu (B.N.P.); kanto.rabema@gmail.com (K.R.); 2 College of Nursing, Armour Academic Center, Rush University, 600 S. Paulina Street, Suite 1080, Chicago, IL 60612, USA; rebekah_hamilton@rush.edu; 3 Lombardi Comprehensive Cancer Center, Georgetown University, 3800 Reservoir Road, NW, Washington, DC 20007, USA; isaacsc@georgetown.edu

**Keywords:** *BRCA1/2*, genetic counseling, intervention development, family support, peer support

## Abstract

Young women from hereditary breast and ovarian cancer (HBOC) families face a series of medical decisions regarding their cancer risk management and integrating this information into their life planning. This presents unique medical and psychosocial challenges that exist without comprehensive intervention. To help lay the groundwork for intervention, we conducted a qualitative study among young women from HBOC families (N = 12; Mean age = 22) and cancer genetic counselors (N = 12) to explicate domains most critical to caring for this population. Women and counselors were interviewed by telephone. The predominant interview themes included preventative care planning and risk management, decision making around the pros and cons of cancer risk assessment, medical management, and psychosocial stresses experienced. Young women endorsed psychosocial stress significantly more frequently than did counselors. Both groups noted the short- and long-term decision making challenges and the support and conflict engendered among familial relationships. Our results suggest young women value the support they receive from their families and their genetic counselors, but additional, external supports are needed to facilitate adaptation to HBOC risk. In feedback interviews focused on intervention planning with a subset of these young women (N = 9), they endorsed the predominant interview themes discovered as important intervention content, a structure that would balance discussion of medical information and psychosocial skill-building that could be tailored to the young women’s needs, and delivery by trained peers familiar with HBOC risk.

## 1. Introduction

Most hereditary breast and ovarian cancer (HBOC) is attributable to a combination of family history and *BRCA1* and *BRCA2* (*BRCA1/2*) mutations, with the latter conferring a 40%–75% lifetime disease risk [1,2]. Many women and men from HBOC families will undergo breast cancer risk assessment/genetic testing (BCRA/GT) with a health care provider to learn their carrier status [3,4,5]. Oftentimes, these carriers have young, first-degree female relatives (i.e., sisters, daughters) at 50% risk of inheriting the familial mutation and who are informed of HBOC risk in the family. Young women then live much of their adolescent and young adult lives knowing that a *specific* disease-causing genetic mutation segregates in their families [6,7]. BCRA/GT for familial mutations provides definitive positive or true negative risk information [8]. Depending on their age and preferences, carriers can manage their HBOC risk through surveillance and/or surgical interventions that greatly reduce the risk of breast and ovarian cancer mortality [9]. Definitive risk information not only informs medical management, but also impacts the timing of important life choices and influences psychosocial adaption. Therefore, BCRA/GT presents young women with the opportunity for cancer risk reduction and adopt a proactive stance to life planning that was largely unavailable to previous generations. These include education, employment, partnering, childbearing, and other tasks of development.

Despite these potential benefits, awareness of genetic predisposition to HBOC at a young age presents specific challenges. Even though they face high lifetime breast cancer risks (approximately 50%), young women with *BRCA1/2* mutations face relatively low 10-year breast cancer risks (1%–2%) [10]. National guidelines in the U.S. call for women to receive an annual breast MRI at age 25–29, with mammography by age 30 [11]. Mutation carriers may consider risk-reducing mastectomy, but are generally recommended to undergo risk-reducing oophorectomy by age 35–40. Thus, plans for risk-reducing surgeries affect future childbearing and breastfeeding. Therefore, BCRA/GT leaves young women with difficult risk management decisions and outcomes requiring them to consider complex tradeoffs between short- vs. long-term health and psychosocial consequences [7]. Cognitive and emotional readiness to reconcile these short- and long-term perspectives simultaneously brings stress to this population and presents clinical challenges. These young women are adjusting to this information at a time when their relationships with immediate family members may be shifting. During emerging adulthood, commonly defined as ages 18–29, young women generally seek greater independence from their parents. HBOC risk, however, may require them to establish closer ties for both information and support [12]. Peers and romantic partners are common sources of support for young adults as well, but often have difficulty appreciating the experience unless they, too, have lived it themselves [13].

Baum’s Model of Stress and Genetic Testing [14] applies Lazarus and Folkman’s theory of stress and coping [15] to the genetic testing context. For the experiences of young women from HBOC families, the model conceptualizes familial HBOC risk as a stressor (i.e., threat) that prompts secondary appraisals of the threat. Coping behaviors include emotional adjustment and behaviors to address this threat and reduce distress. This complex coping process can be further explicated by the Theory of Genetic Vulnerability [16], which suggests that young women from HBOC families are influenced by their familial experience with cancer when thinking about their own future risk of HBOC and subsequent choices for BCRA/GT and risk reduction. Today’s young women may seek to cope with their genetic disease risk in ways that improve upon the outcomes of previous generations--both medically and psychosocially. They have also greater access to information and social connectedness to other young woman at risk [17]. However, we presently lack a complete understanding of young women’s perceptions of BRCA/GT and related interventions that would support them in achieving more positive outcomes.

Given their unique medical and psychosocial needs, model protocols are needed that may serve as adjuncts to young women’s medical care. In the absence of such protocols, we anticipate that both patients and providers struggle with complex HBOC risk management, and the provision of education and counseling that optimizes health and well-being [18]. To lay the groundwork for such endeavors, we conducted a qualitative study among young women from HBOC families and cancer genetic counselors. The purpose was to explicate domains most critical to caring for this population, to identify perceptions of need among patients and counselors, and to assess the initial acceptability of intervention content, format and delivery mode.

## 2. Methods

### 2.1. Participants

Eligible young women were identified by first or second-degree relatives who carried *BRCA1* or *BRCA2*. Index carriers were recruited from research and clinical registries at a comprehensive cancer center and active for research re-contact. Index carriers were male or female, over the age of 21 years, affected or unaffected with cancer, and able to read and understand English. Young female relatives were between the ages of 19–28 years and first- or second-degree relatives of the index carriers and may have previously undergone BRCA/GT.

Eligible young women were contacted by a research assistant (C.E.) after being identified by index carriers with permission for contact. Eligible young women were mailed a letter informing them that they had been identified by index carriers for a study about HBOC and invited to participate and complete a semi-structured qualitative interview. They received a $20 gift card for participating.

Genetic counselors were recruited via email and phone contact with community providers. Eligibility included being a genetic counselor whose practice included or was solely dedicated to clinical cancer genetics. We purposively sampled providers in different geographic regions of the US (Northeast, Southeast, Midwest) practicing in diverse settings (comprehensive cancer centers, hospital- and clinic-based practices). We approached counselors via email in the Fall of 2014. Each counselor received $50 for his/her time.

### 2.2. Interview

We utilized a sequential design for this study. We began with a semi-structured interview for young adult women and genetic counselors, administered by telephone and audiotaped. Interview questions centered around three themes: the influence of family and other social supports (i.e., romantic partner and friends) in adapting to HBOC risk and BRCA/GT, the experience of BRCA/GT, and the implications of HBOC risk and BRCA/GT for life planning. Examples of these questions are provided in Table 1.

We allowed our results from these initial analyses to inform a second, iterative, mixed-methods interview with a subset of the young adult women in our sample. Content of this second interview focused on three domains related to intervention development: (1) the importance of the major themes that had emerged from our data, rating these on a 1–10 scale (1 = not at all important, 10 = exceptionally important); (2) the helpfulness of intervention content focused on medical information, accessing social support, provider communication, and self-efficacy (1 = not at all helpful, 10 = exceptionally helpful); and (3) the overall rating of a program that would focus on these domains (1 = not at all helpful, 10 = exceptionally helpful), their likelihood to join a similar program (Y/N) and the perceived helpfulness of this program to be delivered by a peer coach (1 = not at all helpful, 10 = exceptionally helpful). The opportunity to provide brief open-ended responses to expand on their rating was offered following their ratings, with the exclusion of their ratings of the major themes.

### 2.3. Data Analysis

All interviews were transcribed verbatim, organized and coded using NVivo 10 software. Two coders (C.E. and S.C.O.) assigned thematic codes to the interviews. The themes were compared, definitions agreed upon, with emerging themes added and charts created. These codes were applied to all interviews and refined to include any new themes as they emerged. Responses could be coded to more than one theme and differences in code assignment were resolved by inter-coder discussion. Inter-coder agreement averaged 98% across themes. Descriptive statistics were used to help describe patterns in the coded responses. Analyses were conducted to explore differences by set (young women vs. genetic counselors). The quality of study reporting was assessed using the Consolidated Criteria for Reporting Qualitative Research (COREQ) by the authors (Appendix A). Each response to our mixed-methods interview was compiled calculating a frequency count of each item within tertile ranges (1–3, 4–6, 7–10). All subjects gave their informed consent for inclusion before they participated in the study. This study was conducted in accordance with the Declaration of Helsinki, and the protocol was approved by the Ethics Committee of Georgetown University (2012-1550).

## 3. Results

All young women approached agreed to participate (N = 12). Among the 15 genetic counselors approached, 1 actively declined, and 2 did not respond or were not interviewed due to scheduling conflicts. Interviews continued until data saturation was achieved in both groups. Ultimately, 12 counselors participated. The recruitment of young women in our sample was limited by their index carriers’ participation in a clinical cancer registry that consented to research re-contact.

Interviews lasted on average 15.43 min for young adult women (range 6.14–31.30 min) and 21.24 min for genetic counselors (range 14.48–37.52 min). As seen in Table 2, the 12 young women interviewed were all White (*n* = 12), with a mean age of 23.5 (range = 19–28 years). Seven of the young women had previously been tested for a familial *BRCA1/2* mutation, with 4 participants testing positive. Nearly all counselors were female (*n* = 11/12, 91.7%) and White (*n* = 11/12, 91.7%). With respect to employment settings, 41.6% worked in an academic medical center setting and 33.3% worked in a private medical center or practice. Counselors had been in practice from 1–20 years, with an average of 8.3 years and a median of 6.0 years in practice. All counselors reported working often with HBOC patients.

As seen in Table 3, the predominant interview themes included (1) care planning and management; (2) decision making around pros and cons of testing; (3) medical management; and (4) the psychosocial distress experienced by this population of young women. Means were similar when coding frequencies were divided by the number of participants, with the exception of psychosocial distress: young women noted this more prominently than counselors (t = 2.55, *p* = 0.02). This table also displays the various themes and how they were categorized, found among the interviews with young women and genetic counselors along with the frequency that each theme occurred.

**Figure 1:**
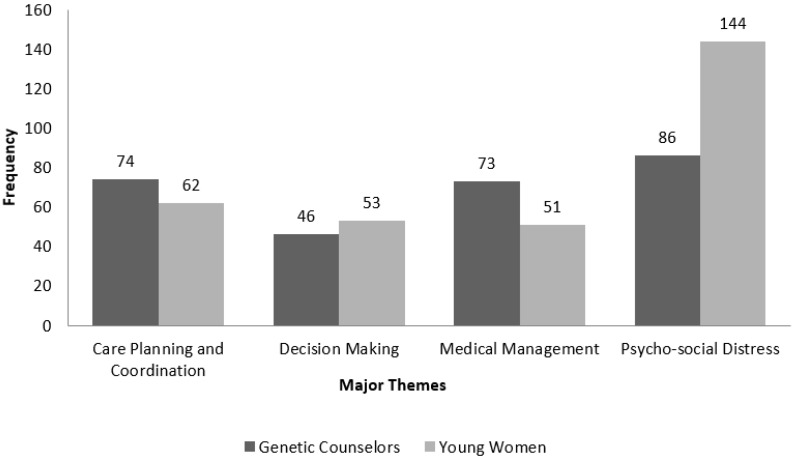
Frequency differences of major themes by group.

Figure 1 illustrates the differences in the predominant interview themes of (1) care planning and management; (2) decision making around pros and cons of testing; (3) medical management; and (4) the psychosocial distress as mentioned by the young women and genetic counselors. This figure shows that among the four themes, decision making and psychosocial distress were noted more by young women than genetic counselors and genetic counselors based more importance on care planning and coordination and medical management.

### 3.1. Care Planning and Management

Young adult women voiced a need to receive clear and direct explanations of their HBOC risk. They wanted a better understanding of BRCA/GT beforehand to establish realistic expectations and receive instruction about follow-up.

“I guess what happens after the test results … getting information about this, how this could affect your life or not, or this is what (the information) could mean”.

-Patient, Age 26, tested positive

Most of the young women expressed a desire to receive all of the information at once and to also have all of the clinical jargon defined and explained to them, instead of having information cut out.

“I’m a person who likes all of the information … and I don’t want it to be dumbed-down. It was really frustrating going to physicians who wouldn’t explain things to me, then also very troubling to get bits and pieces of information over time”.

-Patient, Age 24, untested

They also cited the need to have consistent information or a source for reliable information when they received conflicting recommendations from important sources such as their family and health care providers.

When my mom said, “Oh, you need mammograms, …” and the gynecologist said “I didn’t need them—I would get stressed out”.

-Patient, Age 24, untested

In turn, two primary domains emerged from counselors’ interviews about the genetic counseling and testing process with younger adult women. First, counselors noted that when working with young adult women, they seek to have them consider both the potential value and emotional impact that genetic test results might (or might not) have for them at the present time vs. the value it has for them over the longer term.

“I end up posing a lot more hypothetical questions ... making sure that this is something that they’ve thought through and it’s something that they’re doing for themselves and not due to familial influences”.

-Genetic Counselor

In counseling young women regarding the decision to test, counselors also reported an interest in highlighting that the decision to have testing at a given point in time also opens consideration of additional health decisions that will follow the receipt of a definitive test result.

“So it’s really about being action-oriented … it moves from opening up the emotional can of worms, addressing it, reining it back in, and then being very action-focused is how I tend to handle those consultations”.

-Genetic Counselor

### 3.2. Decision Making about BCRA/GT

The majority of young adult women from HBOC families were learning more about their genetic risks while preparing for entrance into adulthood. For many of them, this was the first decision they made concerning their healthcare, shaping and changing their path and thinking toward adulthood. While all of the young adult women in the study realized the importance of undergoing BRCA/GT and felt the need to be proactive in managing their risk, they, acknowledged their age and stage in life when considering BRCA/GT.

“If (the genetic test) comes back positive, making decisions about how I want to protect myself would be difficult. I think one difficult thing is the timing—when is the best time to make a decision about prophylactic measures”.

-Patient, Age 22, tested (results not in at time of interview)

“I already know that I’m at high risk for (cancer) so I don’t really see any difference between knowing I’m higher risk and knowing I’m positive”.

-Patient, Age 19, untested

Genetic counselors also acknowledged the effect that age has on the decision to undergo BRCA/GT.

“… what we decide at 18, may be very different from what we would decide at 22 or 23. If this is not something that you’re going to act on now, you may feel like you want to know now but if we revisit this in a year or two, you’re going say, ‘Oh gosh, I’m glad I don’t know’. So it’s thinking about how people’s minds can change as their life experiences change”.

-Genetic Counselor

“I think that people have a lot of growth emotionally, and someone might be in a very different head space when they’re in their 20s … so I think that could be a potential like downside. So it’s like the benefit is for them to know earlier and cope with that result and when they’re ready to get screened, they can start. But are you doing harm giving them information that they can’t do anything with for a few years?”

-Genetic Counselor

Counselors also noted the impact of family members on the decision to *undergo BRCA/GT*. Many counselors stated that they see young women who seek counseling and testing to allay the concerns of their family members, often their mothers or sisters. This circumstance also translates to more younger women being less certain in their motivations for testing and how they will use the information in the near future for their own medical or emotional benefit.

“There definitely are women who are doing it for their family but they really have no idea where they’re going in life and this idea of finding out four years before it’s even going to matter—that doesn’t really sit well for some people”.

-Genetic Counselor

“I feel like I have more experiences with younger women coming in and then deciding that they don’t want to do testing. Whereas older women that are coming in, they come in with more of a set idea …. But younger women may not be like that because of just life experience but also that may not be where they are in life”.

-Genetic Counselor

Likewise, the relative lack of experience in life decisions among younger vs. older women can leave them less prepared to consider the downstream consequences of knowing one’s genetic risk for cancer through a confirmed molecular diagnosis. However, despite their reservations, counselors noted that patients should be autonomous to make these decisions.

“I think that the risks are really where they are in their life decision making. And their capacity to actually manage information like this and what we would hope would be normal decision making about finding a partner and whether or not you want to have children and how that can change as a result of being tested. So I think there are risks, but it isn’t for us to decide that they can’t be tested—some people are so concerned that they want to be tested”.

-Genetic Counselor

### 3.3. Medical Management

Both young adult women and counselors noted the importance of fertility issues and plans for risk management in this population (51 vs. 73, respectively in Table 3). While young women seek to preserve fertility, most indicated that their long-range plan was to implement the same risk-reducing measures as their relative.

“I think that I have lived my life basically with an assumption that I would have to get breast removal surgery like my mom did. It was actually interesting to me when one of the physicians was more like, ‘You don’t have anything to worry about now, don’t get tested”. Just the concept of not doing that, was (confusing), I don’t get what you’re saying… And why wouldn’t I have to do that?”

-Patient, Age 24, untested

“I think that knowing that you do have it (a positive test result)—it would give me more clout in negotiating with my physicians when I want to be more aggressive. Just because they say, ‘oh, don’t worry about it, you don’t have to do that yet … ‘and I can say, ‘But wait, I am positive for these mutations, we all know that my risk goes up’.”.

-Patient, Age 24, untested

Despite recognizing the importance of knowing their genetic test results and the utility of testing, most young women didn’t feel the results affected their lifestyle decisions or personal plans for the future. Even for women who adopted screening, healthy eating, and exercise they felt no need to change the pace of life plans involving career goals or romantic relationships.

“I think that even when I’m thinking about life plans now, I don’t take the risk or the genetic testing much into account. I think it would definitely help in making decisions about being realistic about what might happen but right now it doesn’t have much effect on what I’m deciding to do”.

-Patient, Age 20, untested

“Having the information definitely would not change any of my career goals. That’s something that even if I knew I would die tomorrow, I would still plan because there’s only a chance that it (getting cancer) will happen anyways”.

-Patient, Age 19, untested

“I definitely do a lot more screening because of it. I would say as far as day to day life, I haven’t really changed anything. I think with each year, getting a little bit older, I’m a little more cautious about eating healthier and exercising more”.

-Patient, Age 28, tested positive

While counselors reported the importance of providing care that reflects evidence-based guidelines, many also underscored the long-range planning involved in caring for this population. They noted that risk management plans evolve over time and may change throughout a woman’s lifetime and that some of their patients required additional support to consider the long-term implications of test results. Therefore, many sought to not only discuss the patient’s plan for the next steps for medical management, but also to connect them with a set of providers and series of decisions that would unfold in the years to come.

“I try to be very transparent about what national recommendations are. Until they’re 25, these screening recommendations don’t need to go into effect—of course, with the caveat of depending on family history”.

-Genetic Counselor

“In our practice we don’t want people to fall through the cracks. So we set them up either with someone is our practice to be followed once or twice a year or with a breast surgeon if that is who we are talking about. Just let them know that there are options available”.

-Genetic Counselor

“It’s not a single encounter. It is a process, it’s a team initiative working with the physicians and where social work is indicated, behavioral health is indicated”.

-Genetic Counselor

### 3.4. Psychosocial Distress

Regardless of where they were in the process of BRCA/GT (whether tested or untested), young women reported psychological distress as a common reaction.

“I was really nervous … it was a very stressful day. It was a little hard to isolate how I was feeling about that in particular [the test results] but it was a little like, ‘I’m starting my life here, finally out of school’. It was like one of those moments when you’re not invincible anymore”.

-Patient, Age 26, tested positive

“I think that if the results were negative, I think there would be an emotional sort of burden lifted. I feel that burden getting heavier the older I get, the closer I get when my aunts were diagnosed”.

-Patient, Age 24, untested

The data from young adult women and genetic counselors suggested that while family can be a source of distress, they are also a primary source of support. Throughout the interviews with young women, comments were made that suggest their family members are supportive of their choices and decisions.

“I think that being honest is good because … me and my mom’s relationship … it’s very easy to talk to her about that (genetic counseling) and I think that that’s good because it provides more of a security blanket for me that I’m not going into this blind”.

-Patient, Age 20, untested

Further, the young women noted their choice to be tested reflected an autonomous decision, made independently of the influence of their family.

“… it was always my choice and my decision. My family always gave me the power of whether or not I should be tested”.

-Patient, Age 28, tested positive

The genetic counselors reported that they observed a different set of influences. Being able to witness their carrier family member’s experience with testing can be of help to younger women. With that said, many young women appear to follow the example of their family members, without fully considering their own preferences.

“I feel like they already have experienced it, ... they’ve gone through the process so sometimes I think there’s an expectation for these (young) women of what they’re supposed to do. There’s sometimes the element of pressure to meet the expectations of what others have done”.

-Genetic Counselor

Despite this, counselors often mentioned that most young women come to genetic counseling appointments accompanied by an older family member, usually a mother, to provide support. Their presence can help young women better understand the process, know what to expect, and serve as a support system to show that survivorship is possible, mitigating stress.

“I think in the end, it was good that she was there to support her sister, that she didn’t go through it alone and now she gets to watch her sister and see because they both ended up testing positive”.

-Genetic Counselor

“… the family is really in more of a support role, an extra set of ears … they are often taking notes”.

-Genetic Counselor

Further, counselors note that many younger patients present with higher levels of distress than they see in their older patients. As providers, they feel that their job is to empower the patient first, as for many of them this is their first time making a major health decision, in addition to providing emotional management.

“... from a behavioral perspective there is much more emotional counseling in that young group, having to make complex decisions that are harder than someone who may be much older”.

-Genetic Counselor

### 3.5. Intervention Development

Following the ascertainment of these themes, we attempted to recontact the young women for a brief mixed-methods interview to inform intervention development, reaching a majority of the sample (N = 9). The interview gathered feedback on the acceptability of a potential intervention involving telephone counseling and education sessions with a peer coach. As seen in Table 4, young adult women strongly endorsed the importance of the major themes that had emerged from our data. Seven out of nine young women strongly endorsed three of the four themes. The medical management theme was strongly endorsed by eight out of nine women. Session content also was strongly endorsed by the participants. All of the young women strongly endorsed the overall program, and seven of nine strongly endorsed a peer-coaching model.

## 4. Discussion

Young adult women from HBOC families face difficult, complex decisions about BCRA/GT and follow-up, often making important health decisions for the first time in their lives. Four predominant themes emerged in our interviews as important foci for intervention development, including care planning and management, decision making around pros and cons of testing, medical management, and psychosocial distress. With limited information aside from the prior experience of the family as a guide, confronting one’s own cancer risk and approaching this BCRA/GT can result in distress. This distress can hamper the ability to create a long-term plan for medical management [19]. Framed through Baum’s model of stress and genetic testing [14] and the Theory of Genetic Vulnerability [16], these experiences could be altered in this population through more effective coping resources that allow women to engage in problem-solving and social support.

The most notable contrast between the responses of young adult women and counselors was in the area of psychosocial distress. While both young women and genetic counselors noted that the higher level of distress among young adult women is one of the unique aspects of counseling in this group, young women noted this theme significantly more often. The main predictors of long-term distress in women who have undergone BCRA/GT for HBOC is premorbid distress and younger age [20]. We know that distress can interfere with health decision making [21], underscoring the importance of effectively managing distress early in the counseling and testing process. Results of recent systematic reviews [22,23] suggest that several subgroups of tested and untested at-risk women are at risk for heightened distress and current distress screening protocols used in practice are not sufficient in serving their specific needs.

While existing social support through the family can prove positive for young women, family support can lead to more distress and altered perceptions of risk. Previous work has highlighted navigating these family dynamics as a primary challenge for genetic counselors working with this population and their families [18]. Our study shows that in addition to empowering young women, there is also the possibility that it may influence them to pursue testing, consider the same risk management plan followed by prior affected generations, and discourage them from seeking out their own options. Young women are holding their family experience at the forefront as they contextualize their understanding of their risk and the choices they will make to avoid it. Our results suggest that these women could benefit from additional social supports and intervention.

These emotional outcomes meet behavioral outcomes through the impact on care planning and management. In this domain, young adult women and counselors presented paralleled but contrasting views. Meeting with a genetic counselor may be one of the only times young adult women have to receive neutral, educational information specifically pertaining to their risks. Counselors’ descriptions of their goals for their work with this population reflect the unique developmental needs of these young women. Genetic counselors in our sample also noted that rather than confronting management decisions that are often imminent for older patients, long-term management and care planning are crucial for these women. The decision making process for young women with HBOC risk is prolonged, usually not requiring immediate intervention and varies as their life plans change. Further, they tend to seek out information and make decisions based on their needs at the present time. For example, young women often delay their risk management decisions for more than a year after testing [19]. Further, medical options will change over time, as options for risk management become a more viable option as they age. Given these decisions could occur long after the genetic counseling consult, counselors emphasized the value of providing anticipatory guidance for their young patients [24,25]. This can allow young women to consider what they can expect to face in the years to come and to plan accordingly.

In addition to the anticipatory guidance for medical management, counselors also highlighted the use of techniques to support young women in considering their future feelings about cancer risk and risk management. Given their limited experience with health decision-making, their heightened distress and the lasting consequences of knowing one’s risk, counselors highlighted their prompting of their patients to consider the value of the genetic information for them in the present and how this information will impact them emotionally. This technique is well-captured by the use of affective forecasting, or projecting one’s future emotional state. Imagining the future in this way can be challenging, but can allow young women to make predictions about their emotional responses to possible future decisions that may come about from receiving a positive or negative test result. This also helps young women discern what is important to them and make decisions on whether to uptake various screening or preventative measures within the context of their risk and personal value system at their current stage of life. Affective forecasting could not only help young women to consider their decisions, but can alert genetic counselors to the desires of the patient, further guiding decision making [26].

Young adult women strongly endorsed the content, structure and modality of a tailored telephone counseling intervention delivered by an expert peer coach working with a care manual. Support through an informed peer could serve as a means to integrate the personal and emotional supports of family and the expert guidance offered by genetic counselors, but do so in a way that meets the unique developmental needs to this population. Additional research is needed to test the acceptability and feasibility of delivering this manualized care. However, such an intervention could be used to reach a population of young women who are often geographically separate from their carrier relatives. This modality would also overcome time- and access-related barriers that young adult women likely face.

Results of this qualitative study underscore the important role that cancer genetic counselors play in the adaptation of young women from HBOC families. Given the relative gap in the emphasis placed on psychosocial distress by young women vs. counselors, those who work with this population could consider directly assessing the current emotional state of the women they see in their practice and discuss the potential impact significant distress can have on health decision making and, when appropriate, provide referral for additional care. This issue of distress is being examined within a larger quantitative study to further examine its impact on BRCA testing.

Numerous interventions target BRCA1/2 carriers to reduce distress and support decision making [27,28,29,30,31,32]. However, these trials have recruited women who are, on average, almost 20 years older than the women in our study. Therefore, the unique developmental needs of this population likely were not fully addressed. These would include levels of distress and the series of decisions that would face this population in the coming years. Our preliminary results support acceptability of an intervention tailored to the decisions currently facing the young woman and prepare her for the decisions that follow. The majority of young women in high-risk families learn about their genetic risks between the ages of 17 and 20 from their family members and the degree of family history impacts their perceived risk [33,34]. As a result, young women with personal experiences closely tied to family legacies of illness and loss because of cancer are the most likely to seek cancer genetic services and resources for managing cancer risk to avoid family experience with the disease and alter their own personal experience [13].

Our results suggest that young women value the support that they receive from their families and their genetic counselors, but that additional supports addressing their unique needs is warranted and welcome [7]. Trained peer coaches who face hereditary cancer risk, have been educated on the topic and follow manualized treatment protocols of the four sessions we detailed. They could provide a unique source of information and emotional support. Peer support programs are common in the oncology setting [35,36] and preventative medicine and could offer young women a different, but complementary, resource that is not present among their families or care teams [37]. Telephone counseling-based interventions for *BRCA1/2* carriers have demonstrated positive effects in reducing distress and information needs [27,32], though, again, have recruited older women and addressed the needs of women who are imminently deciding about risk-management strategies and coping with the impact of results on partners and children. While younger women might also be facing these issues, there are additional needs that have gone unmet and deserve greater intervention.

While our study brings together the perspectives of young women and cancer genetic counselors, our study has some limitations. Our sample of young women was insured and not ethnically or geographically diverse so our results may not generalize to more heterogeneous populations and involved a small number of participants. Likewise, while our genetic counselors represented diversity of practice setting and time in practice, they were also primarily White and female. We also did not include providers from other specialties who often counsel women about testing decisions (ob/gyn, oncology). Additional insights about risk management could have been available. Our qualitative methods did not address questions that quantitative methods could have addressed, such as level of distress. We will describe levels of distress following the completion of a survey that is currently in the field.

## 5. Conclusions

These results provide unique insights about the design of interventions for young women from HBOC families. Future interventions for this population should include the familial nature of risk and communication and coping skills training targeted at the uncertainty faced by this population. Guidance from genetic counselors and providers should be tailored to meet their individual risk with particular consideration to their desires with continual follow-ups as they manage their risk.

## Figures and Tables

**Table 1:** Interview guide.

Interview Content		
**Interview Themes**	**Genetic Counselors**	**Young Women**
**Role of the Family and Social Support in Testing**	What role does the family play in counseling? Partner, friends, outside resources?	Who started the conversation and when? What did you think the genetic test results meant for your family member’s health? Was your family member available to discuss genetic testing if you had any questions? Have they brought up the topic of you undergoing testing/counseling? How did your family, friends, providers play a role?
**Counseling and Testing Process**	Do women vary in whether they are ready for testing at this age? How is counseling different for young women that have a familial cancer risk? What are the risks/advantages of testing young women? What concerns do you have about testing in this age group?	Do you plan to get testing in the future? When? What prompted you to move ahead with testing? Did you seek out additional resources?
**Implications of Testing**	What techniques do you use when talking to women about testing? How do you present and resolve the issue of fewer risk management options for younger women?	How do you think this information has changed or will change your personal life plans? Having children? Long-term relationships? Do you think knowing the information will be helpful to you?

**Table 2:** Participant characteristics.

Participant Variables	Young Women (N = 12)	Genetic Counselors (N = 12)
	M (SD) N (%)	M (SD) N (%)
**Gender**		
Female	12 (100)	11 (91.7)
Male		1 (8.3)
**Race/Ethnicity**		
White	12 (100)	11 (91.7)
African American		1 (8.3)
**Age**	22.6 (2.84)	35.4 (7.94)
**Work Setting**		
Academic medical center		5 (41.6)
Private group practice		4 (33.3)
Government		3 (25.1)
**Years in Practice**		8.3 (6.41)

**Table 3:** Code frequencies for response theme

Themes	Coding Sets	Individual Nodes	Genetic Counselors	Young Women
Care planning and coordination			74	62
	Intervention		70	32
		Technique	39	11
		In print resources	0	4
		Decision support	4	12
		Long Term Cancer Risk	7	3
		Short Term Cancer Risk	11	1
		Affective forecasting	9	1
	Clinical communication		4	30
		Young women-talking to providers	0	24
		Results communication-how delivered	0	3
		Negative Result-initial reaction	0	3
		Rapport with young patients	4	0
Decisions related to pros and cons			46	53
	Harms of testing		27	24
		Life insurance	3	11
		Harms of testing	4	4
		GINA	2	2
		Genetic discrimination	2	0
		Confusion about what to do with results	0	1
		Age of testing-potentially too young	16	6
	Benefits of testing		19	29
		Young women-testing effects romantic relationships	2	3
		Work planning	4	5
		Benefits of testing	13	21
Medical management			73	51
	Fertility		23	11
		Surgical menopause	1	0
		Fertility and family planning	22	11
	Risk Management		50	40
		Young women-future plans for management	0	25
		Surgery	11	7
		Screening	22	2
		Deciding Not to be tested	10	1
		Healthcare navigation	7	5
Psycho-social Distress			86	144
	Familial Influences		45	115
		Young women-sibling discussion	0	10
		Young women-finding out about family risk	0	28
		Testing influences	16	16
		Family Support	4	3
		Presence of parent changes counseling	8	0
		Family distress	12	5
		Negative family communication	2	0
		Young women sharing results	0	11
		Family communication	1	26
		Positive family communication	2	16
	Peers/Partners		20	7
		Group support	8	1
		Friend support	1	0
		Romantic partner support	3	0
		Online support	4	3
		Feelings of isolation	4	3
	Patient Distress		21	22
		Patient distress	21	22

**Table 4:** Frequency of Acceptability of Intervention Content, Structure and Modality (N = 9).

Domain	1–3 N (%)	4–6 N (%)	7 + N (%)	Sample Quote
Theme 1: Care Planning and Management	0 (0%)	2 (22%)	7 (78%)	n/a
Theme 2: Decision Making about BCRA/GT	0 (0%)	2 (22%)	7 (78%)	n/a
Theme 3: Medical Management	0 (0%)	1 (11%)	8 (89%)	n/a
Theme 4: Psychosocial Distress	0 (0%)	2 (22%)	7 (78%)	n/a
Session 1: Information about genetic cancer risks, medical management; how to make medical choices consistent with values/preferences	0 (0%)	2 (22%)	7 (78%)	“It’s something that’s hard to make yourself look for on your own. To have it put together would make it more likely that someone would (use it)”.
Session 2: Coping skills; learning where/how to reach out for help	0 (0%)	0 (0%)	9 (100%)	“Even people [who] know how to deal in general with their own stress and anxiety … I think it is a different way of coping that we may not realize we don’t really know how to deal with”.
Session 3: Skill building for medical management and patient-provider communication.	0 (0%)	1 (11%)	8 (89%)	“Helping you to be more of a partner in your medical decisions. I think it would be helpful … as far as what to do now that I have my results”.
Session 4: Review of skills gained in Session 1–3, focused on self-efficacy in implementing skills.	0 (0%)	1 (11%)	8 (89%)	“It’s very reinforcing, and it’s a good review to make sure that everything is going to be good going forward”.
Overall Program Rating	0 (0%)	0 (0%)	9 (100%)	“It sounds like it would be very helpful to young women going through this, and helping decide what they’re going to do, calming them down about it, and making sure that they know they have this communication with their doctor”.
Endorsement of a Peer Coach Model	1 (11%)	1 (11%)	7 (78%)	“It would provide that extra layer of support and uniqueness to the program. To talk to somebody who is in similar age group, and recently went through a similar decision and situation would be really helpful”.

## Appendix A. COREQ Checklist

**No.**	**Item**	**Guide Questions/Description**	**Response**
**Domain 1: Research Team and Reflexivity**			
Personal Characteristics			
1.	Interviewer/facilitator	Which author (s) conducted the interview or focus group?	Dr. Suzanne O’Neill conducted the interviews with genetic counselors and Chalanda Evans conducted the interviews with the young women.
2.	Credentials	What were the researcher’s credentials? e.g., PhD, MD	- Suzanne C. O’Neill, PhD, Associate Professor in the Department of Oncology. - Chalanda Evans, BS, Project Director. - Rebekah J. Hamilton, PhD, RN, FAAN, Associate Professor in the Women, Children and Family Nursing Dept. - Kenneth Tercyak, PhD, Associate Professor in the Dept. of Oncology and Pediatrics. - Beth N. Peshkin, MS, CGC, Professor of Oncology and Senior Genetic Counselor at Lombardi Comprehensive Cancer Center. - Kantoniony Rabemananjara, BS Candidate, Research Intern.
3.	Occupation	What was their occupation at the time of the study?	
4.	Gender	Was the researcher male or female?	All researchers but Kenneth Tercyak were female.
5.	Experience and training	What experience or training did the researcher have?	- Drs. O’Neill and Tercyak are clinical psychologists who have conducted research in this area for many years. - Dr. Hamilton and Ms. Peshkin also have conducted research in this area for many years, as well as providing clinical care to this population. - Ms. Evans has pursued mixed-methods training beyond that provided in her Bachelor’s degree program. She has several years of experience managing Dr. O’Neill’s research program in genomics. - Ms. Rabemananjara has a Bachelor’s degree and research experience in Dr. O’Neill’s lab.
Relationship with participants			
6.	Relationship established	Was a relationship established prior to study commencement?	None of the young women was acquainted with the researchers prior to the study start. Some of the genetic counselors were professional colleagues of Dr. O’Neill through professional organizations.
7.	Participant knowledge of the interviewer	What did the participants know about the researcher? e.g., personal goals, reasons for doing the research	The participants knew the purpose of the interview was to hear more about their opinions and thoughts regarding issues of genetic testing, cancer prevention, and family health for young women, aged 18–30 at risk for carrying a BRCA1/2 mutation. Participants were made aware the researchers were affiliated with Lombardi Comprehensive Cancer Center and the interview was part of a larger NCI funded study.
8.	Interviewer characteristics	What characteristics were reported about the interviewer/facilitator? e.g., bias, assumptions, reasons and interests in the research topic	
**Domain 2: Study Design**			
Theoretical framework			
9.	Methodological orientation and theory	What methodological orientation was stated to underpin the study? e.g., grounded theory, discourse analysis, ethnography, phenomenology, content analysis	Grounded theory
Participant selection			
10.	Sampling	How were participants selected? e.g., purposive, convenience, consecutive, snowball	- Genetic counselors were recruited using a purposive sample to recruit for diversity in geographical and practice setting, followed by a snowball sample from these initial participants. - Young women were recruited consecutively from women participating in a quantitative study until saturation was met.
11.	Method of approach	How were participants approached? e.g., face-to-face, telephone, mail, email	Participants were approached by telephone to participate after completing an initial qualitative survey.
12.	Sample size	How many participants were in the study?	24 total (12 genetic counselors and 12 young women).
13.	Non-participation	How many people refused to participate or dropped out? Reasons?	One counselor who was approached refused participation due to competing commitments. No individuals agreed but then dropped out before the interview.
Setting			
14.	Setting of data collection	Where was the data collected? e.g., home, clinic, workplace	Interviews were conducted by telephone at Georgetown University, Lombardi Comprehensive Cancer Center.
15.	Presence of non-participants	Was anyone else present beside the participants and researchers?	No one else was present while researchers conducted the interview and to our knowledge all participants completed the interviews in a private setting with no else present.
16.	Description of sample	What are the important characteristics of the sample? e.g., demographic data, date	Age, gender, level of education, BRCA testing status
Data collection			
17.	Interview guide	Were questions, prompts, guides provided by the authors? Was it pilot tested?	A semi-structured interview guide was developed. The guides were modified as needed to fit the participant being interviewed. The guides were not tested in a pilot study.
18.	Repeat interviews	Were repeat interviews carried out? If yes, how many?	Yes, repeat interviews were carried out with XX of the participants to ask follow up questions.
19.	Audio/visual recording	Did the research use audio or visual recording to collect the data?	Audio recording was used for all interviews and transcribed prior to analysis.
20.	Field notes	Were field notes made during and/or after the interview or focus groups?	Yes, during the phone interviews.
21.	Duration	What was the duration of the interviews or focus group?	- Genetic counselor interviews—21.54 min approx. - Young women interviews—15.43 min approx.
22.	Data saturation	Was data saturation discussed?	Yes. Interviews with genetic counselors and young women ended when saturation was reached.
23.	Transcripts returned	Were transcripts returned to participants for comment and/or correction?	No. Transcripts were reviewed by researchers who listened to the audio recordings to verify their accuracy.
**Domain 3: Analysis and Findings**			
Data analysis			
24.	Number of data coders	How many data coders coded the data?	2 (SO and CE)
25.	Description of the coding tree	Did authors provide a description of the coding tree?	Yes, a coding tree was developed based upon the individual nodes, coding sets and themes from the data.
26.	Derivation of themes	Were themes identified in advance or derived from the data?	Initial coding was informed by the interview guide but codes were continually refined and created with data collection and analysis. All codes were agreed upon by the coders thru consensus.
27.	Software	What software, if applicable, was used to manage the data?	QSR NVivo version 10
28.	Participant checking	Did participants provide feedback on the findings?	No
Reporting			
29.	Quotations presented	Were participant quotations presented to illustrate the themes/findings? Was each quotation identified? e.g., participant number	Yes, participant quotations are present but are not identified by participant number.
30.	Data and findings consistent	Was there consistency between the data presented and the findings?	Yes
31.	Clarity of major themes	Were major themes clearly presented in the findings?	Yes
32.	Clarity of minor themes	Is there a description of diverse cases or discussion of minor themes?	Yes
